# Aortic coarctation: guidelines mismatch across the ocean

**DOI:** 10.1186/1749-8090-9-38

**Published:** 2014-02-20

**Authors:** Martino Pepe, Fortunato Iacovelli, Filippo Masi, Vito Marangelli, Arnaldo Scardapane, Alessandro De Santis, Luca Sgarra, Donato Quagliara, Stefano Favale

**Affiliations:** 1 Section of Cardiovascular Diseases, Emergency and Transplantations Department, “Aldo Moro” University, Piazza Giulio Cesare 11, 70124 Bari, Italy; 2 Section of Radiology, Interdisciplinary Department of Medicine, “Aldo Moro” University, Piazza Giulio Cesare 11, 70124 Bari, Italy

**Keywords:** Aortic coarctation, Computed tomography, Guidelines, Pseudocoarctation

## Abstract

Pseudocoarctation is a rare congenital anomaly characterized by aorta elongation and kinking, without significant obstruction. We report the case of an elderly patient with history of congestive heart failure (CHF) and aortic regurgitation (AR) who was referred for progressive exertional dyspnoea. After multimodal imaging evaluation, aortic coarctation with significant trans-stenosis gradient but mild luminal narrowing was diagnosed; this borderline patient was not addressed to repair, according to ESC guidelines and in spite of AHA ones. He rather met the criteria for pseudocoarctation diagnosis. An integration of functional and anatomical data is essential for a reliable diagnostic process in similar cases.

## Background

Coarctation of the aorta (CoA) is a congenital malformation consisting in a narrowing of the aorta, usually located at the isthmus, and generating a pressure gradient between the aortic arch and the distal thoracic aorta. Clinical appearance of severe CoA is usually in the early adulthood or earlier; 90% of untreated patients die before reaching the age of 50 years. Hypertensive vascular complications, cerebrovascular hemorrhages, aortic aneurysm, aortic dissection or rupture, aortic valve deterioration, premature coronary disease are the most frequent life-threatening complications.

Consequently the key of the diagnostic/therapeutic process is an accurate assessment of the malformation severity and of the ideal timing for intervention.

## Case presentation

**Figure 1:**
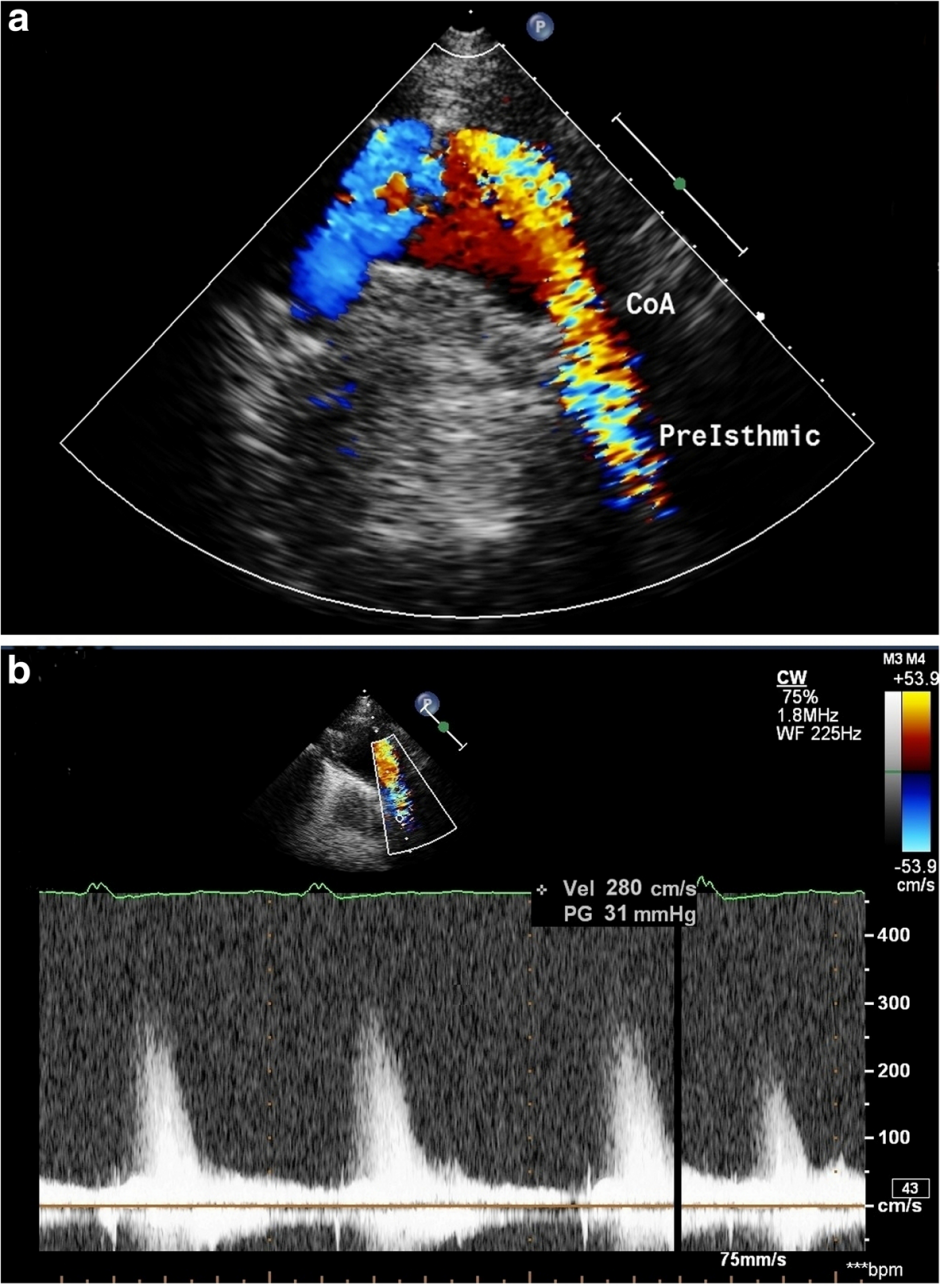
**Transthoracic echocardiography through dorsal approach.** Color Doppler flow acceleration across the CoA **(a)**; significant trans-stenosis pressure gradient **(b)**.

**Figure 2:**
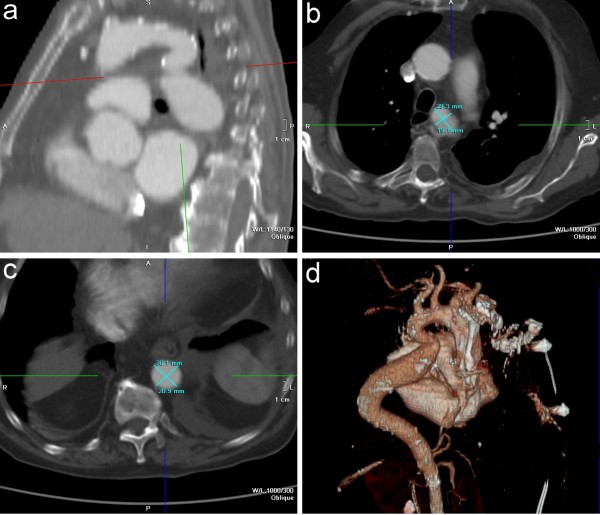
**Computed Tomography angiography.** 2D parasagittal scan view of CoA **(a)**; 2D CoA diameters **(b)**, 2D aortic diameters at the diaphragm level **(c)**; 3D volume-rendered reconstruction of the thoracic aorta showing significant tortuosity and no developed collateral circulation **(d)**.

We present the case of a 77-year-old Caucasian man admitted to our division for worsening of exertional dyspnoea and left pleural effusion superimposed upon a 10 years long history of hypertension (current good pharmacological control), permanent atrial fibrillation and CHF. Anamnesis indicated a New York Heart Association (NYHA) functional class III (despite optimal medical therapy) while physical examination highlighted a faint 2^nd^ heart sound, a 3^rd^ heart sound and an ejection systolic murmur audible at the interscapular region; no significant pressure gradient between upper and lower limbs was detected. Echocardiography showed severe AR within a normal tricuspid valve anatomy, aortic root dilatation (40 mm), left ventricle (LV) dysfunction (ejection fraction 40%) and, through a dorsal approach, a flow acceleration across a kinked segment of the descending aorta (maximum pressure gradient of 31 mmHg) with a post-stenosis mild ectasia (diameter of 38 mm) (Figure 1). Angiography revealed a non-obstructive coronary artery disease and a kinked aorta with a narrowing at the isthmic segment developing a trans-stenosis peak-to-peak systolic gradient of 25 mmHg with a proximal arterial pressure of 150/80 mmHg; as an additional movie file shows in more detail (Additional file 1), no collateral circulation was revealed. CT scan confirmed the above mentioned anatomic data and allowed precise intraluminal measures: the CoA cross sectional diameters were 29 × 19 mm compared to reference diameters at the diaphragm level of 30 × 29 mm (Figure 2).

Cardio-surgery consult indicated surgical repair of the aortic valve and no treatment for the CoA. The patient declined any intervention and was discharged with medical therapy prescription. At 1 year follow-up he satisfies II NYHA functional class with no further intercurrent hospitalizations.

## Discussion

Current ESC guidelines do not include trans-stenosis pressure gradient among the pro-intervention parameters [1]; indications for repair are a non-invasive pressure difference > 20 mmHg between upper and lower limbs or hypertension associated with a ≥ 50% narrowing relative to the reference aortic diameter (diaphragm level). Our patient did not match either the arterial pressure or the anatomic criteria, thus was correctly not addressed to surgical repair of the CoA.

This case raises some interesting issues: the lack of uniformity among the international guidelines on this topic and the undefined role of co-morbidities (such as AR) in the severity assessment of the disease. According to current AHA guidelines patients with a peak-to-peak pressure gradient > 20 mmHg (as it was in our patient) satisfy the first criterion for interventional/surgical repair recommendation [2]. As a result to date a rigorous adherence to guidelines would make the same patient differently treated in Europe and North America.

For sure functional parameters (i.e. invasive pressure gradient) have the advantage to reflect the real pathophysiological effect of an anatomical anomaly; nevertheless they are rather difficult to be interpreted. In our case, for example, the co-presence of severe AR can be easily misleading. No guidelines help physicians in this task. In our patient it is reasonable to hypothesize that severe AR increases the pre-load and the related stroke volume (SV); this raise pushes the trans-CoA pressure gradient to higher levels than those expected in relation to the anatomic narrowing. Conversely the reduced Ejection Fraction decreases the SV causing an opposite effect on pressure gradient and making its interpretation and the decision-making process more complex.

Moreover also strictly anatomical parameters can be misleading: the relative narrowing of the isthmic aorta (compared to diaphragmatic diameter) can be biased by an extensive aortic disease, mainly in older patients. Thus Takeda recently proposed a novel “cross sectional area” cut-off indexed to the patients’ body surface as a possible alternative indicator of CoA severity [3]. Anyway, in the presented case, even when indexed to our patient’s body surface (2.1 m^2^), anatomical parameters did not reach threshold for intervention indication.

These considerations suggest the need for an accurate “case by case” evaluation. In the reported case anatomical data and patient’s age at symptoms presentation support the conclusion of a “non severe” CoA despite the pressure gradient degree (probably influenced by AR). This case would better match the recently proposed “aortic pseudocoarctation” definition, usually characterized by lower increase of the LV after-load (as compared to “true CoA”) and milder pathophysiological effects such as the absence of collateral circulation [4].

## Conclusions

Further studies are anyway needed to establish whether functional rather than anatomical parameters are the gold standard to assess the disease severity and to clarify the way by which functional data should be interpreted in the presence of misleading co-morbidities, either cardiac (e.g. LV dysfunction, aortic valve disease) or not cardiac (e.g. anemia). It is authors’ opinion that integration of functional and anatomic data could be the best to reach a reliable diagnostic process and that uniformity in Western Countries’ guidelines is required.

## Consent

Written informed consent was obtained from the patient for publication of this Case report and any accompanying images. A copy of the written consent is available for review by the Editor-in-Chief of this journal.

## Competing interests

The authors declare that they have no competing interests.

## Authors’ contributions

AD, LS, VM and AS collected the data. MP and FI interpreted the data and drafted the manuscript. FM and DQ revised critically the manuscript. SF managed a critical revision of the article and finally approved the manuscript. All authors read and approved the final manuscript.

## Supplementary Material

Additional file 1 **Aortography.** Aortic root dilatation with severe AR, and significant tortuosity of the whole thoracic aorta with isthmic kinking. Click here for file
